# The Chronic Kidney Disease Epidemiology Collaboration equations perform less well in an older population with type 2 diabetes than their non-diabetic counterparts

**DOI:** 10.3389/fpubh.2022.952899

**Published:** 2022-08-10

**Authors:** Shimin Jiang, Danyang Zhang, Wenge Li

**Affiliations:** ^1^Department of Nephrology, China-Japan Friendship Hospital, Beijing, China; ^2^Graduate School of Peking Union Medical College, Peking Union Medical College and Chinese Academy of Medical Sciences, Beijing, China

**Keywords:** CKD-EPI equations, glomerular filtration rate, type 2 diabetes, elderly, creatinine, cystatin C

## Abstract

**Objectives:**

The Chronic Kidney Disease Epidemiology Collaboration (CKD-EPI) equations are based on creatinine alone (CKD-EPIcr), cystatin C alone (CKD-EPIcys) and combined creatinine and cystatin C (CKD-EPIcr-cys). It remains unclear whether these equations perform differently in older adults with type 2 diabetes than they do in non-diabetic older individuals.

**Methods:**

This single-center cross-sectional study was performed in adults aged ≥ 65 years between January 2019 and December 2021. Glomerular filtration rate (GFR) was measured by technetium-99m-diethylene triamine pentaacetic acid (^99m^Tc-DTPA) renal dynamic imaging. The bias (difference between measured and estimated GFR), precision [interquartile range (IQR) of the median difference between measured GFR and estimated GFR] and accuracy P30 (percentage of estimated GFR within 30% of measured GFR) were considered the criteria of equation performance.

**Results:**

Finally, 476 participants were enrolled, including 243 adults with type 2 diabetes and 233 non-diabetic adults. The mean age of the included participants was 71.69 ± 6.4 years and 262 (55%) were male. The mean measured GFR was 49.02 ± 22.45 ml/min/1.73 m^2^. The CKD-EPIcr-cys equation showed significantly greater bias and lower accuracy (P30) in individuals with diabetes than in the non-diabetic group (median bias, 4.08 vs. 0.41 ml/min/1.73 m^2^, respectively, *p* < 0.05; P30, 63.78% vs. 78.54%, respectively, *p* < 0.05). The precision IQR indicated that CKD-EPIcr-cys had also lower precision in individuals with diabetes than in the non-diabetic controls (17.27 vs. 15.49 ml/min/1.73 m^2^, respectively). Similar results were observed for CKD-EPIcr and CKD-EPIcys equations. The P30 of all three equations failed to reach 80% in diabetic and non-diabetic groups.

**Conclusions:**

The performance of the CKD-EPI equations was lower in a group of patients aged ≥ 65 years with type 2 diabetes than in non-diabetic counterparts. However, each equation still had limitations regarding accuracy in older adults with or without diabetes.

## Introduction

The number of people older than 65 years with diabetes worldwide was 135.6 million in 2019, and is projected to increase to 276.2 million by 2045 (1). The prevalence of diabetes in adults has more than tripled over the last two decades, from an estimated 151 million (4.6% of the global population) in 2000 to 537 million (10.5%) today (2, 3). The aging of the population and the increase in the prevalence of diabetes are two important factors associated with the increased incidence of chronic kidney disease (CKD) (4). Precise estimation of glomerular filtration rate (GFR) is critical for diagnosis, classification, and management of patients with CKD, particularly in those with comorbid diabetes (5).

The GFR is regarded as the best overall index of kidney function in both health and disease. However, measurement of GFR using clearance of inulin (6), iohexol (7), or ^125^I-iothalamate (8) is invasive and may be too inconvenient and costly for use in everyday practice. Technetium-99m-diethylene triamine pentaacetic acid (^99m^Tc-DTPA) renal dynamic imaging (9), which is recommended for GFR measurement by the Nephrology Committee of the Society of Nuclear Medicine (10), has been widely used in clinical practice. However, as ^99m^Tc-DTPA is also inconvenient, the use of GFR-estimating equations has become more common.

In 2009, the CKD Epidemiology Collaboration (CKD-EPI) initially developed a GFR-estimating equation based on serum creatinine for use in individuals with and without kidney function loss (11). Given the association between aging and physiological changes in the kidneys as well as the potential effects of muscle mass on serum creatinine, the CKD-EPI then developed two other equations based on cystatin C alone and in combination with creatinine in 2012, and demonstrated that the combined creatinine-cystatin C equation had better performance than equations based on either of these markers alone (12). However, the applicability of these equations in older Chinese adults with diabetes is unknown. This study was conducted to assess the performance of three CKD-EPI equations in a population of older individuals with type 2 diabetes in comparison with their non-diabetic counterparts.

## Patients and methods

### Study design

This case-control study was conducted at China-Japan Friendship Hospital, Beijing, China, between January 2019 and December 2021. Participants, who were diagnosed with acute kidney failure, receiving dialysis, or with dehydration or fluid overload were excluded. Type 2 diabetes was diagnosed according to the 2022 American Diabetes Association (ADA) criteria (13).

### Data collection and measurements

Clinical information, including laboratory (serum levels of creatinine, cystatin C and albumin) and demographic data (age, sex, and disease history) were obtained from the Electronic Medical Record System of our center. Serum creatinine level was determined by enzymatic kinetic assay under fasting conditions, and cystatin C was measured using a latex particle-enhanced turbidimetric immunoassay. Patients' heights and weights were also recorded.

The reference GFR was measured using ^99m^Tc-DTPA renal dynamic imaging. The results were normalized to a body surface area (BSA) of 1.73 m^2^, as described by the Dubois method: BSA (m^2^) = 0.007184 × body weight (kg)^0.425^ × height (cm)^0.725^ (14).

### CKD-EPI equations

The eGFR was calculated using the Creatinine Equation (CKD-EPIcr 2009) (11), Cystatin C Equation (CKD-EPIcys 2012), and Creatinine-Cystatin C Equation (CKD-EPIcr-cys 2012) (12). The CKD-EPIcr equation (2009) is expressed as follows: 141 × min(Scr/κ, 1)^α^ × max(Scr/κ, 1)^−1.209^ × 0.993^age^[× 1.018 if female] [× 1.159 if Black], where Scr is serum creatinine, κ is 0.7 for females and 0.9 for males, α is −0.329 for females and −0.411 for males, min is the minimum of Scr/κ or 1, and max is the maximum of Scr/κ or 1 (11). The CKD-EPIcys equation (2012) is expressed as follows: 133 × min(Scys/0.8, 1)^−0.499^ × max(Scys/0.8, 1)^−1.328^ × 0.996^age^ [× 0.932 if female], where Scys is serum cystatin C, min indicates the minimum of Scys/κ or 1, and max indicates the maximum of Scys/κ or 1 (12). The CKD-EPIcr-cys equation (2012) is expressed as follows: 135 × min(Scr/κ, 1)^α^ × max(Scr/κ, 1)^−0.601^ × min(Scys/0.8, 1)^−0.375^ × max(Scys/0.8, 1)^−0.711^ × 0.995^age^ [× 0.969 if female] [×1.08 if Black], where Scr is serum creatinine, Scys is serum cystatin C, κ is 0.7 for females and 0.9 for males, α is −0.248 for females and −0.207 for males, min indicates the minimum of Scr/κ or 1, and max indicates the maximum of Scr/κ or 1 (12).

### Statistical analysis

Three criteria were considered in the evaluation of equation performance: bias, precision, and accuracy. Bias was expressed as the median difference (MD) between measured GFR (mGFR) and eGFR, where a negative or positive bias indicated overestimation or underestimation of eGFR, respectively. Precision was expressed as the interquartile range (IQR) of the difference between mGFR and eGFR. Accuracy was considered under two criteria: root mean square error (RMSE), defined as the square root of the average squared difference of eGFR and mGFR on a logarithmic scale; and P30, defined as the percentage of estimates within 30% of mGFR. When P30 is > 90%, the equation fulfills the requirements of clinical interpretation (6, 15, 16). By using the Wilcoxon, McNemar, and χ^2^ tests, differences between equations were compared. Bland-Altman analysis was performed to examine the agreement between mGFR and eGFR. The smaller the width between 95% limits of agreement (LOA), the better agreement. Statistical analyses were conducted using SPSS (version 23.0; IBM Corp., Armonk, NY, USA), and MedCalc (version 20.0.15; MedCalc, Mariekerke, Belgium). Statistical significance was defined as a value of *p* < 0.05.

## Results

### Characteristics of the study population

**Figure 1:**
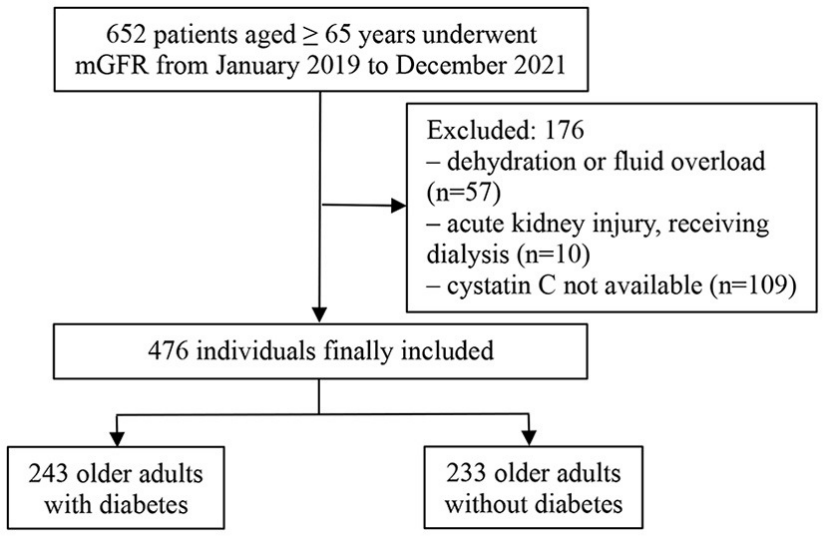
Flowchart of the study patients. mGFR, measured glomerular filtration rate; PSM, propensity score matching.

Of an initial 652 older adults, 476 fulfilled the study criteria (Figure 1), 262 (55%) of whom were male. The mean age of the participants was 71.69 ± 6.4 years. The mean mGFR was 49.02 ± 22.45 ml/min/1.73 m^2^. Participants were divided according to the presence or absence of type 2 diabetes into the diabetic group (243 participants) and non-diabetic group (233 participants). Table 1 shows the demographic and main laboratory data of the participants. Older adults with diabetes had significantly lower mGFR than the non-diabetic group (46.17 ± 23.3 vs. 51.99 ± 22.28 ml/min/1.73 m^2^, respectively, *p* = 0.005). In addition, diabetic participants had a slightly higher level of body mass index than those without diabetes (25.08 ± 3.03 vs. 24.35 ± 3.34 kg/m^2^, respectively, *p* = 0.027). However, there were no significant differences in age, sex, and serum albumin between the two groups (Table 1).

**Table 1:** Demographic and clinical data for participants aged 65 years and older^*^.

	**Overall (*n* = 476)**	**Individuals with diabetes (*n* = 243)**	**Individuals without diabetes (*n* = 233)**	***p***
age, years	71.69 ± 6.40	71.54 ± 6.31	71.84 ± 6.51	0.60
males, *n* (%)	262 (55.0)	130 (53.5)	132 (56.7)	0.49
BMI, kg/m^2^	24.72 ± 3.20	25.08 ± 3.03	24.35 ± 3.34	0.027
serum albumin, g/L	38.88 ± 4.87	38.57 ±5.19	39.21 ± 4.48	0.16
serum creatinine, mg/dl	1.91 ± 1.79	2.08 ± 1.80	1.74 ± 1.76	<0.05
serum cystatin C, mg/L	1.87 ± 1.13	2.04 ± 1.20	1.70 ± 1.01	0.001
mGFR, ml/min/1.73 m^2^	49.02 ± 22.45	46.17 ± 23.30	51.99 ± 22.28	0.005
eGFR, ml/min/1.73 m^2^				
CKD-EPIcr	49.71 ± 26.08	45.48 ± 25.51	54.12 ± 26.0	<0.001
CKD-EPIcys	45.67 ± 25.70	41.49 ± 24.74	50.02 ± 26.01	<0.001
CKD-EPIcr-cys	47.01 ± 25.53	42.74 ± 24.74	51.47 ± 25.63	<0.001

* *Data are presented as means and standard deviations, and counts (n) and percentages (%)*.

*BMI, body mass index; mGFR, measured glomerular filtration rate; eGFR, estimated glomerular filtration rate; CKD-EPI, Chronic Kidney Disease Epidemiology*.

### Performance of equations in individuals with or without diabetes

Table 2 shows the performance of the three equations in individuals with and without type 2 diabetes, determined by calculating the bias, precision and accuracy. In the overall population, the bias of CKD-EPIcr was −0.81 ml/min/1.73 m^2^, which was smaller than each of CKD-EPIcys (3.91 ml/min/1.73 m^2^) and CKD-EPIcr-cys (2.24 ml/min/1.73 m^2^). Regarding accuracy P30, only CKD-EPIcr-cys exceeded 70%, which was significantly higher than either CKD-EPIcr (66.81%) or CKD-EPIcys (64.91%). In other words, CKD-EPIcr had the smallest bias in the overall population, but CKD-EPIcr-cys achieved the better precision and accuracy.

**Table 2:** Performance of the three equations in individuals with and without diabetes.

	**Bias**	**Precision**	**Accuracy**	
	**Median**	**IQR (P25, P75)**	**P30**	**RMSE**
Overall (*n* = 476)
CKD-EPIcr	−0.81	18.75 (−9.78, 8.97)	66.81	0.176
CKD-EPIcys	3.91^a^	18.63 (−5.75,12.88)	64.91	0.172
CKD-EPIcr-cys	2.24^a^, ^b^	17.75 (−6.57,11.18)	71.01^a^, ^b^	0.167
Individuals with diabetes (*n* = 243)
CKD-EPIcr	1.26^*^	18.88 (−8.33, 10.55)	62.55^*^	0.199
CKD-EPIcys	5.51^a^^*^	18.51 (−4.02, 14.49)	60.08^*^	0.194
CKD-EPIcr-cys	4.08^a^, ^b^^*^	17.27 (−4.59, 12.68)	63.78^*^	0.193
Individuals without diabetes (*n* = 233)
CKD-EPIcr	−1.22	16.99 (−10.93, 6.06)	71.24	0.147
CKD-EPIcys	2.90^a^	17.82 (−6.96, 10.86)	69.96	0.145
CKD-EPIcr-cys	0.41^a^, ^b^	15.49 (−7.10, 8.39)	78.54^a^, ^b^	0.135

*P30 represents the proportion of estimated glomerular filtration rate (GFR) within 30% of measured GFR*.

*IQR, interquartile range; RMSE, root mean square error, the square root of (log measured GFR – log of estimated GFR)2; CKD-EPI, Chronic Kidney Disease Epidemiology*.

a *p < 0.05 vs. CKD-EPIcr in the same group of individuals*.

b *p < 0.05 vs. CKD-EPIcys in the same group of individuals*.

* *p < 0.05 vs. corresponding equations used in individuals without diabetes*.

In individuals with diabetes, the median bias between CKD-EPIcr (1.26 ml/min/1.73 m^2^) and each of CKD-EPIcys (5.51 ml/min/1.73 m^2^) and CKD-EPIcr-cys (4.08 ml/min/1.73 m^2^) was significant (less bias in the former); there was also significant difference in median bias between CKD-EPIcys and CKD-EPIcr-cys (4.08 ml/min/1.73 m^2^). Precision IQR (P75–P25) demonstrated that the CKD-EPIcr-cys equation had higher precision (17.27 ml/min/1.73 m^2^) than CKD-EPIcr and CKD-EPIcys equations (18.88 and 18.51 ml/min/1.73 m^2^, respectively). The differences in accuracy (P30) between the three CKD-EPI equations were not statistically significant (62.55, 60.08, and 63.78%, respectively). CKD-EPIcr, CKD-EPIcys, and CKD-EPIcr-cys had similar RMSE values (0.199, 0.194, and 0.193, respectively). The aforementioned results indicate that the three equations had the similar precision and accuracy although CKD-EPIcr had the smallest median bias.

In individuals without diabetes, the CKD-EPIcr-cys equation showed the lowest bias, and the highest precision and accuracy. The biases of CKD-EPIcr and CKD-EPIcr-cys were −1.22 and 0.41 ml/min/1.73 m^2^, respectively, which were significantly different. Meanwhile, CKD-EPIcys had higher bias than CKD-EPIcr (2.9 vs. −1.22 ml/min/1.73 m^2^, respectively, *p* < 0.05). Precision IQR showed that CKD-EPIcr-cys had the highest precision (15.49) and CKD-EPIcys had the lowest precision (17.82). With regard to accuracy, CKD-EPIcr-cys had higher P30 (78.54%) and lower RMSE (0.135) than each of CKD-EPIcr (71.24% and 0.147, respectively) and CKD-EPIcys (69.96% and 0.145, respectively), although the differences were not significant.

### Comparison of the performance of equations between individuals with and without diabetes

As shown in Table 2, the performance of CKD-EPIcr-cys was less accurate in the diabetic group than in the non-diabetic group. Regarding bias, the median bias of CKD-EPIcr-cys in the diabetic group was significantly higher than in the non-diabetic group (4.08 vs. 0.41 ml/min/1.73 m^2^, respectively, *p* < 0.05). Regarding precision, CKD-EPIcr-cys had the lower in the diabetic group than in non-diabetic group (17.27 vs. 15.49 ml/min/1.73 m^2^, respectively). Regarding accuracy, CKD-EPIcr-cys was less accurate in the diabetic group; it had the lower P30 and higher RMSE in the diabetic group than in the non-diabetic group (P30, 63.78% vs.78.54%; RMSE, 0.193 vs. 0.135). Similar results were observed for CKD-EPIcr and CKD-EPIcys equations.

### Bland-Altman plots of the three equations compared to mGFR

**Figure 2:**
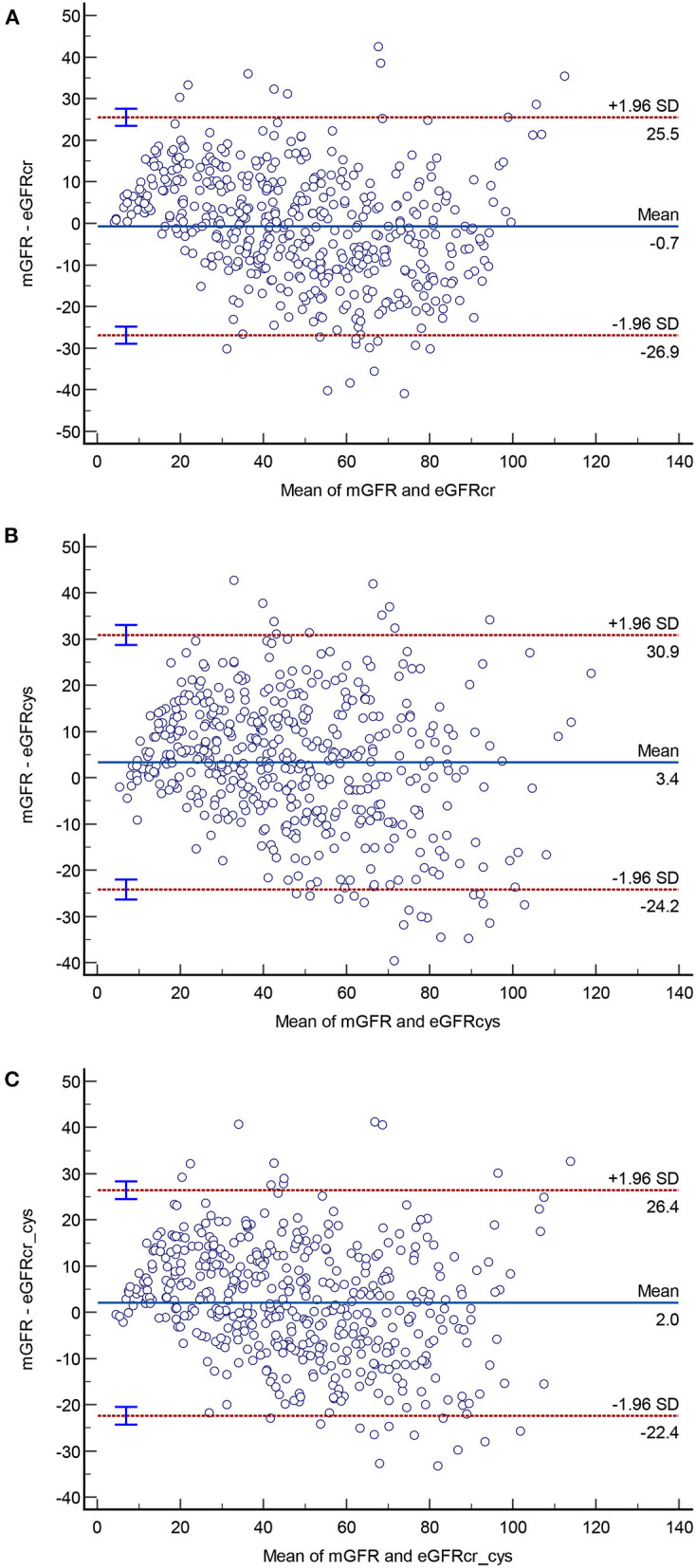
Bland-Altman plots of the three equations **(A–C)** in the overall population.

**Figure 3:**
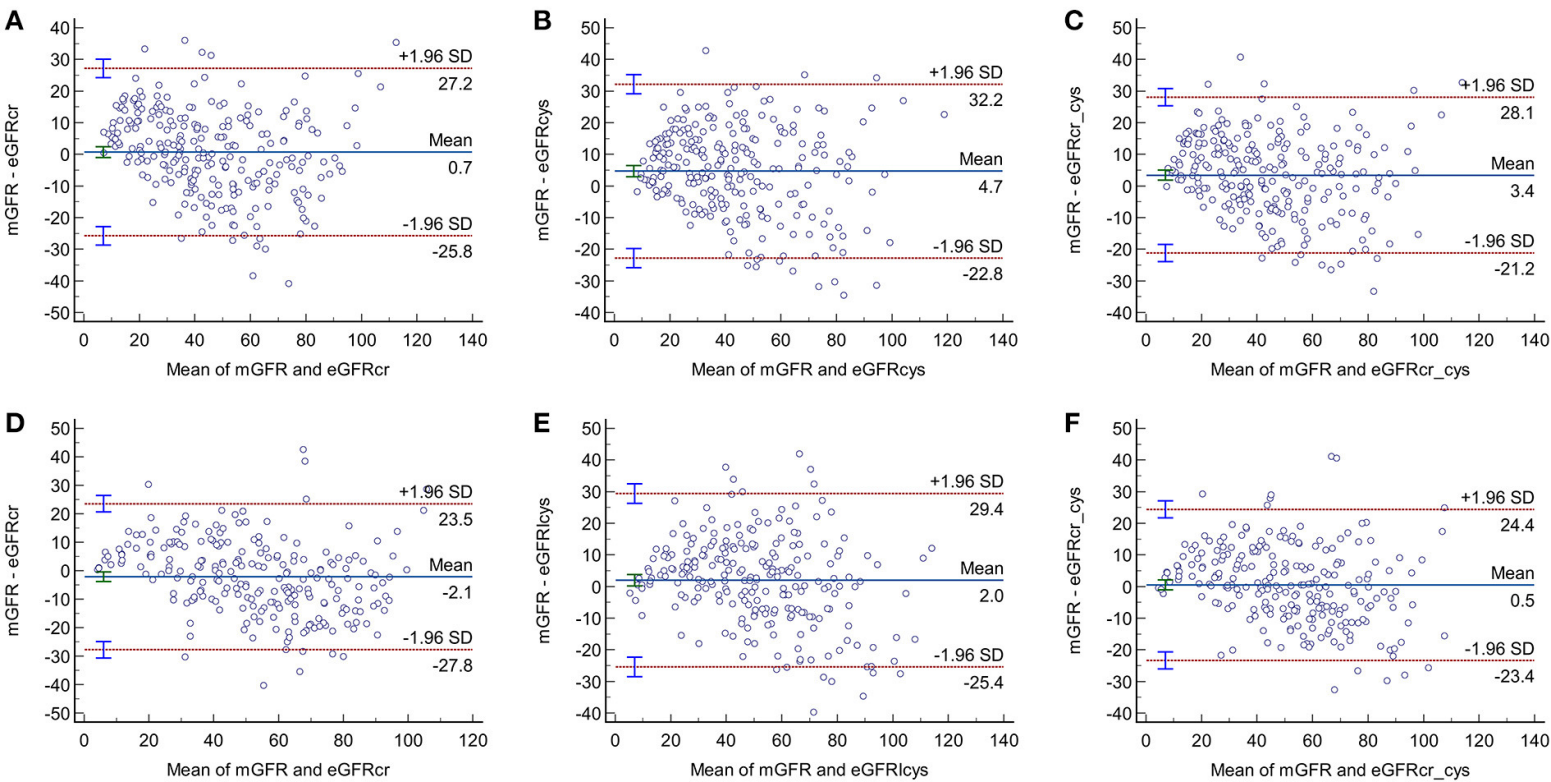
Bland-Altman plots of the three equations in older adults with **(A–C)** and without diabetes **(D–F)**.

Bland-Altman plots of the three equations in the overall population and in persons stratified by diabetes were presented in Figures 2, 3. In the overall population, Bland-Altman analysis showed that CKD-EPIcr-cys had the best agreement; it had the lowest gap between the 95% LOA (CKD-EPIcr, 52.4 ml/min/1.73 m^2^; CKD-EPIcys, 55.1 ml/min/1.73 m^2^; CKD-EPIcr-cys, 48.8 ml/min/1.73 m^2^) (Figures 2A–C).

As shown in Figures 3A–F, in participants with and without diabetes, the gaps between the 95% LOA of the three equations were higher in the diabetic group than in the non-diabetic group (CKD-EPIcr, 53 vs. 51.3 ml/min/1.73 m^2^, respectively; CKD-EPIcys, 55 vs. 54.8 ml/min/1.73 m^2^, respectively; CKD-EPIcr-cys, 49.3 vs. 47.8 ml/min/1.73 m^2^, respectively), suggesting that the consistency of these equations is lower in older subjects with diabetes than in their non-diabetic counterparts.

## Discussion

We evaluated the performance of three CKD-EPI equations in a group of older adults with type 2 diabetes in comparison with non-diabetic counterparts. This study found that the CKD-EPI equations were less reliable in estimating GFR in older adults with type 2 diabetes than in the non-diabetic group. In addition, CKD-EPIcr-cys had the least bias and the best precision and accuracy in adults without diabetes. However, there seemed to be no performance advantages in using any of these equations in diabetic counterparts, although the median bias of CKD-EPIcr was relatively small.

In actual clinical practice, determination of GFR is an important step in assessing renal function. The ADA recommends annual screening for diabetic kidney disease by assessing urinary albumin excretion and GFR (17). The modification of diet in renal disease (MDRD) equation, which was developed in CKD patients, tends to be less accurate than CKD-EPI in those with GFR ≥ 60 ml/min/1.73 m^2^ (11, 18). Thus, other equations were developed, such as CKD-EPI equation. The Kidney Disease Improving Global Outcomes (KDIGO) guidelines recommend use of the CKD-EPI equations to estimate GFR in adults of any age (19), which were developed in a North American and European population (11, 12). In addition to age and sex, these equations also take race into account. Although the proportion of patients aged ≥ 65 years with the CKD-EPI development data sets was 13%, previous study has found that CKD-EPI works satisfactorily in older adults with varying levels of GFR (20).

Older adults typically show a decrease in GFR, and this group is increasing in importance due to the gradual aging of the population (1). However, there have been few studies regarding the application of CKD-EPI equations in adults aged ≥ 65 years (6, 21, 22). As diabetes can induce renal damage and decrease GFR, it is necessary to clarify whether the CKD-EPI equations are equally applicable in older Chinese adults with and without type 2 diabetes.

In this study, GFR was measured using ^99m^Tc-DTPA renal dynamic imaging, which was proposed by the Nephrology Committee of the Society of Nuclear Medicine (9). This method has been widely accepted as applicable for clinical evaluation of renal function (23, 24). Therefore, GFR obtained by ^99m^Tc-DTPA renal dynamic imaging in this study was chosen as the reference GFR.

As muscle mass is frequently reduced in older adults, while plasma cystatin C is less affected, we explored the performance of CKD-EPI equations based on cystatin C alone and in combination with creatinine in older individuals with and without diabetes. All three equations showed a clinically poorer performance, with greater degrees of bias, lower precision, and lower accuracy, in older adults with diabetes than in non-diabetic controls. Our results were similar to a previous study by Camago et al. (25) in a population of 56 adult patients with type 2 diabetes and 55 healthy volunteers in whom the CKD-EPIcr equation was shown to be less accurate in the diabetic group compared to the non-diabetic controls. In a previous study, in a population of 215 diabetic and 192 non-diabetic CKD patients with a broad range of ages, Xie et al. (26) reported that CKD-EPIcr-cys showed the best performance among the CKD-EPI equations, and that eGFR equations were less accurate in the diabetic group than in the non-diabetic group. As these studies did not specifically focus on older adults, we then investigated four creatinine-based equations in people aged ≥ 65 years and our results suggested that the accuracy of creatinine-based GFR-estimating equations was lower in individuals with diabetes (21). However, further investigations are required to determine whether addition of diabetes can improve the performance of CKD-EPI equations in the older population.

On the other hand, the P30 of all three equations in the study failed to reach 80% in the elderly with or without diabetes, suggesting that these equations have limitations regarding accuracy in these populations. P30 exceeding 90% indicates that the equation meets the requirements for clinical interpretation (6, 15, 16). However, care is required in interpreting P30 decline in older adults, as small errors may still indicate inconsistent equations in those with low GFR (mean mGFR < 60 ml/min/1.73 m^2^).

In addition, CKD-EPIcr overestimated GFR and CKD-EPIcys underestimated GFR whether in the overall population or in elderly subjects without diabetes. It may be caused by non-GFR determinants. Lower serum creatinine levels in older adults are often due to lower muscle mass and reduced protein intake. This is because lower muscle mass and reduced protein intake in older adults may lead to a decrease in serum creatinine levels, and the inflammatory status may lead to increased serum cystatin C levels. And in people with diabetes, all equations overestimated GFR and had greater biases, which appeared to be affected by glucose levels, although not fully explainable. Another misconception about the source of serum creatinine may be that a higher body mass index in the diabetic group would be an indicator of higher muscle mass. In fact, it indicates body fat buildup, not muscle mass (27).

This study had several limitations, as the sample size was relatively small sample size and from a single institution. Therefore, further studies with larger populations are required to verify our findings. In this study, GFR was measured by ^99m^Tc-DTPA renal dynamic imaging, and not by inulin clearance. As inulin requires continuous infusions and repeated blood collection, it is not typically used in clinical settings. Finally, since this study is retrospective, some non-GFR determinants (e.g., C-reactive proteins) are incomplete.

## Conclusion

In summary, the CKD-EPI equations were less reliable in estimating GFR in older adults with type 2 diabetes than in their non-diabetic counterparts. In adults with type 2 diabetes, there seemed to be no performance advantages in the use of any of these equations, albeit CKD-EPIcr had the least bias. However, in non-diabetic people, CKD-EPIcr-cys achieved optimal performance among the three equations. Nevertheless, there were still limitations regarding accuracy regardless of the presence or absence of type 2 diabetes. In older adults, especially in those with diabetes, early referrals for CKD treatment may decrease mortality, hospitalization rates, and rates of catheter use for dialysis.

## Data availability statement

The original contributions presented in the study are included in the article, further inquiries can be directed to the corresponding author.

## Ethics statement

The studies involving human participants were reviewed and approved by the Ethics Committee of China-Japan Friendship Hospital. Written informed consent for participation was not required for this study in accordance with the national legislation and the institutional requirements.

## Author contributions

SJ contributed to conception and design of the study, and wrote the first draft of the manuscript. SJ and DZ collected the clinical data. WL revised the final version and was the guarantor of this work. All authors contributed to the article and approved the submitted version.

## Funding

This work was supported by grants from Science and Technology Project of Beijing (D171100002817002), National Key Clinical Specialty Capacity Building Project (2019-542), and National Key R&D Program of China (2018YFC1704304).

## Conflict of interest

The authors declare that the research was conducted in the absence of any commercial or financial relationships that could be construed as a potential conflict of interest.

## Publisher's note

All claims expressed in this article are solely those of the authors and do not necessarily represent those of their affiliated organizations, or those of the publisher, the editors and the reviewers. Any product that may be evaluated in this article, or claim that may be made by its manufacturer, is not guaranteed or endorsed by the publisher.
